# Dielectric imaging for differentiation between cancer and inflammation *in vivo*

**DOI:** 10.1038/s41598-017-13545-3

**Published:** 2017-10-13

**Authors:** Rimi Lee, Sun-Mi Lee, Hyung Joon Kim, Sook Young Kim, Mina Son, Jun Ho Song, Khulan Lkhamsuren, In Ho Park, In Hong Choi, Young Nyun Park, Jeon-Soo Shin, Kyung-Hwa Yoo

**Affiliations:** 1 Graduate Program for Nanomedical Science and Technology, Yonsei University, Seoul, 03722 Republic of Korea; 2 Department of Microbiology, Yonsei University College of Medicine, Seoul, 03722 Republic of Korea; 3 Department of Pathology, Yonsei University College of Medicine, Seoul, 03722 Korea; 4 Department of Physics, Yonsei University, Seoul, 03722 Republic of Korea; 5 Severance Biomedical Science Institute and Institute for Immunology and Immunological Diseases, Yonsei University College of Medicine, Seoul, 03722 Republic of Korea

## Abstract

In this study, we develop an *in vivo* dielectric imaging technique that measures capacitance using pin-type electrode arrays. Compared to normal tissues, cancer tissues exhibit higher capacitance values, allowing us to image the cancer region and monitor the chemotherapeutic effects of cancer in real-time. A comparison with the histopathological results shows that the *in vivo* dielectric imaging technique is able to detect small tumors (<3 mm) and tumor-associated changes. In addition, we demonstrate that cancer and inflammation may be distinguished by measuring the capacitance images at different frequencies. In contrast, the positron emission tomography using 2-[^18^F]-fluoro-2-deoxy-D-glucose was not capable of discriminating between cancer and inflammation.

## Introduction

Positron emission tomography using 2-[^18^F]-fluoro-2-deoxy-D-glucose (^18^F-FDG PET) has been widely employed in cancer diagnosis, distant metastasis staging, and therapeutic monitoring because ^18^F-FDG is accumulated at many malignancies owing to Warburg effect^1,2^. However, the uptake of ^18^F-FDG is not cancer-specific. ^18^F-FDG is also accumulated in inflammation and infection^3,4^, so it is not easy to distinguish cancer tissues from inflamed ones solely on the basis of ^18^F-FDG PET imaging. ^18^F-FDG PET is generally combined with structural diagnostic images such as magnetic resonance imaging (MRI), computed tomography (CT), and ultrasound (US).

The dielectric properties (or capacitance) of tissues have been investigated for several decades since Fricke and Morse first reported different capacitance values for breast cancer and normal tissues in 1926^5^. Compared to healthy tissues, cancer tissues show a substantially increased capacitance, which may be ascribed to changes in membrane properties, cellular contents, and the amount of extracellular fluid^6–9^. More recently, electrical impedance tomography (EIT) has been developed as a non-invasive medical imaging technique to identify specific tissue anatomy and pathophysiology. EIT has a wide spectrum of possible applications, including screening for breast cancer, monitoring for lung or heart problems, and monitoring brain function^10–13^. However, to reconstruct EIT images from surface bioimpedance measurements, complicated non-linear inverse problems should be solved. Moreover, the resolution of EIT is poor in comparison with other imaging techniques, such as CT and MRI.

Here, we present an *in vivo* dielectric imaging technique with 10 × 10 electrode arrays, in which a capacitance image can be acquired without solving inverse problems. Using this capacitance-based *in vivo* imaging technique, we can detect even a tumor with a diameter less than 3 mm. In addition, this capacitance-based *in vivo* imaging technique can be applied to differentiate between cancer and inflammation, and to monitor chemotherapeutic effects in real-time.

## Results

### Capacitance imaging of cancer

**Figure 1:**
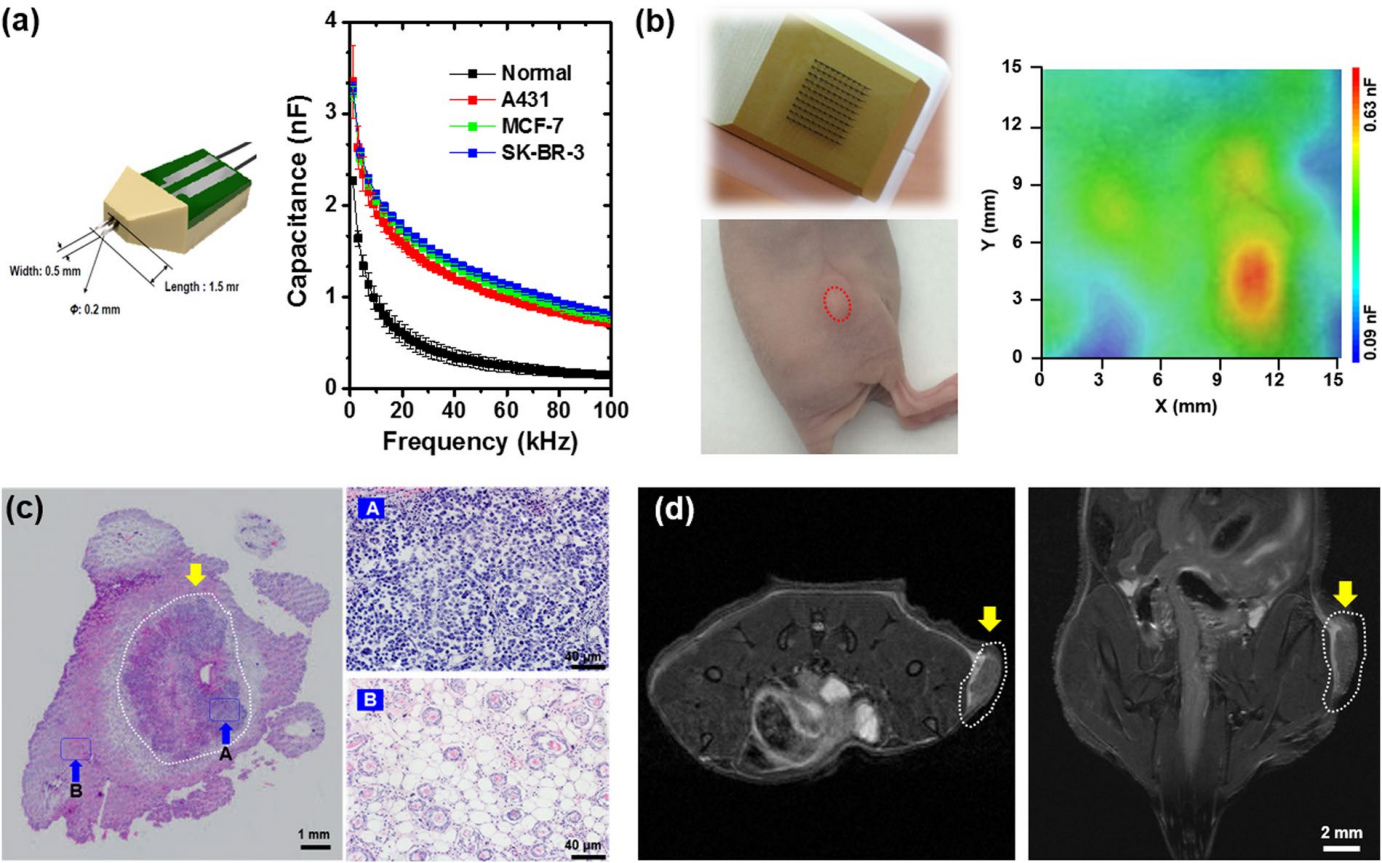
(**a**) (Left) Schematic diagram of probe I spaced 0.5 mm apart. (Right) Frequency dependence of the capacitance measured for normal and cancer tissues from MCF-7, SK-BR-3, and A431 tumor-bearing mice. Data represent mean ± standard deviation (*n* = 5). (**b**) (Left, top) Image of probe II composed of 10 × 10 electrodes 1.5 mm in length and spaced 0.5 mm apart, and fabricated on a flexible substrate. (Left, bottom) Image of an SK-BR-3 tumor-bearing mouse (red dotted area). (Right) Capacitance image of the cancer region on a 50-color scale. The 9 × 9 capacitance values were measured using probe II with an AC voltage of 10 mV at 100 kHz. The color range was set between 0.09 and 0.63 nF with the red color denoting the highest capacitance. (**c**) Hematoxylin and eosin (H&E)-stained histological specimens obtained from cancer tissue (white dotted area) extracted from the SK-BR-3 tumor-bearing mice after the capacitance measurement. The right column shows the higher magnification of histological images of the blue dotted rectangles denoted by (top) A and (bottom) B. (**d**) MRI image of an SK-BR-3 tumor-bearing mouse.

We first constructed a pin-type probe (probe I); the pins of length 1.5 mm spaced 0.5 mm apart (Fig. 1a). Figure 1a shows the frequency dependence of the capacitance measured using probe I with a 10-mV peak-to-peak AC voltage for normal or cancer tissues of SK-BR-3, MCF-7 (both human breast cancer cell lines), and A431 (a human epidermoid carcinoma cell line) tumor-bearing nude mice; data were obtained from five mice and then averaged. We observed higher capacitance values for cancer tissues than for normal tissues over the entire frequency range, as reported previously^6,8^. These findings suggested that the cancer region may be imaged by measuring the capacitance values.

**Figure 2:**
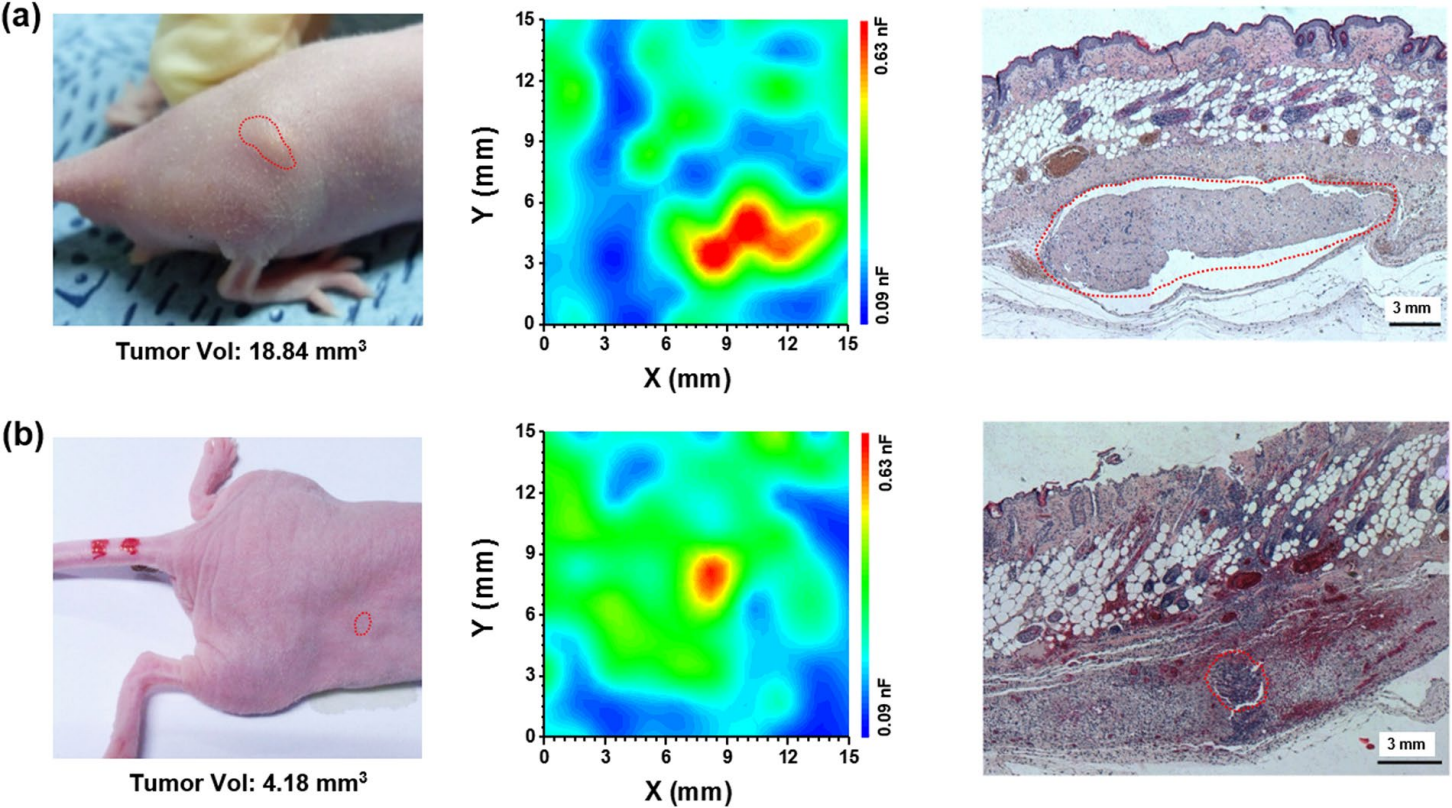
(Left) Image of SK-BR-3 tumor-bearing mice showing different tumor volumes, as indicated by the red dotted area. The tumor volumes were (**a**) 18.84 mm^3^ and (**b**) 4.18 mm^3^. (Middle) Capacitance images of cancer regions on a 50-color scale for (**a**) large- and (**b**) small- sized cancers. A 9 × 9 capacitance array was measured using probe II with an AC voltage of 10 mV at 100 kHz. The color range was set between 0.09 and 0.63 nF, with the red color denoting the highest capacitance. (Right) H&E-stained histological specimens of cancer tissues extracted from the SK-BR-3 tumor-bearing mice with (**a**) large- and (**b**) small-sized cancers after the capacitance measurements. When compared with the histological results, the red region in the capacitance images corresponds to the cancer region (red dotted area).

For capacitance imaging, a probe array, composed of 10 × 10 pin-type electrodes 1.5 mm in length placed 0.5 mm apart (probe II), was fabricated on a flexible substrate. We measured the capacitance at 100 kHz using each electrode pair in the cancer region of SK-BR-3 tumor-bearing mice, and we plotted 9 × 9 capacitance values on a 50-color scale using the software OriginPro® version 9, where the red region denotes the highest capacitance (Fig. 1b). A comparison with the histopathological results (Fig. 1c) and the MRI images of the mouse tumors (Fig. 1d) revealed that the red region corresponded to the cancer region. Outside of the cancer region, no red regions were observed (Fig. S1b). We also measured the capacitance image at 100 kHz for other SK-BR-3 tumor-bearing mice with different tumor volumes and shapes (Fig. 2a,b), and found that subcutaneous tumors with a diameter near 3 mm could be imaged.

**Figure 3:**
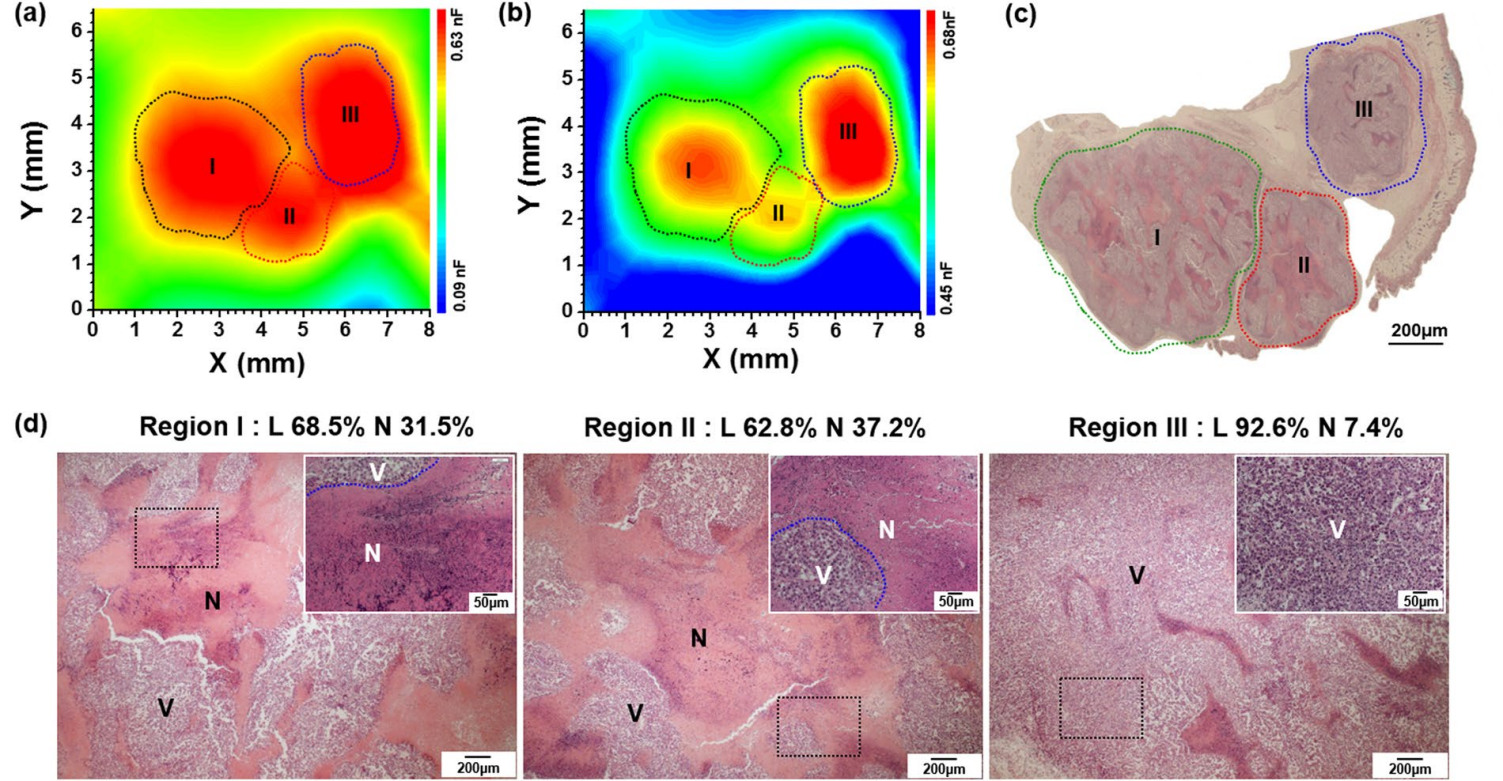
Capacitance images measured with an AC voltage of 10 mV at 100 kHz for the cancer region, as obtained from the SK-BR-3 tumor-bearing mouse with a relatively large-sized cancer (38 mm^3^) on different capacitance scales: (**a**) 0.09 ~ 0.63 nF and (**b**) 0.45 ~ 0.63 nF. (**c**) H&E-stained histological specimens of cancer tissues extracted from the SK-BR-3 tumor-bearing mouse after the capacitance measurements. (**d**) Histopathologic sections of regions I, II, and III. Most tissues in regions I and II are necrotic, whereas tumor tissues in region III are viable without necrosis. The insets show the higher magnification microscopic features marked by black dotted rectangles. Vor L: Viable region, N: Necrotic region. The capacitance images in 15 × 15 mm are shown in Fig. S2.

Figure 3a illustrates the capacitance image obtained from an SK-BR-3 tumor-bearing mouse with a relatively large-sized cancer volume of 38 mm^3^. When this capacitance image was plotted on a scale of 0.09 ~ 0.63 nF, we observed three cancer regions (I, II, and III) in red color (Fig. 3a). However, when this capacitance image was replotted on a narrower range of 0.45 ~ 0.63 nF for better resolution of regions I-III, regions I and II changed color to orange and yellow, respectively, while region III remained red. This indicated that the capacitance of region I or II was lower than that of region III; the average capacitance values were 0.638 ± 0.025, 0.587 ± 0.031, and 0.698 ± 0.090 nF for regions I, II, and III, respectively (Fig. 3b). According to histopathological data, the tumors in regions I and II were necrotic in 31.5% and 37.2%, respectively, and had a relatively scarce cell density; however, the tumor in region III had high cell density with a high viable cancer cell area (92.6%) (Fig. 3c,d). Central necrosis inside a tumor is an extreme representative manifestation of hypoxia^14^. Therefore, the lower capacitance in regions I and II may be attributed to more extensive necrosis within cancer tissues, suggesting that the necrotic area within cancer tissues may be monitored using the capacitance imaging method.

### Discrimination between cancer and inflammation imaging

**Figure 4:**
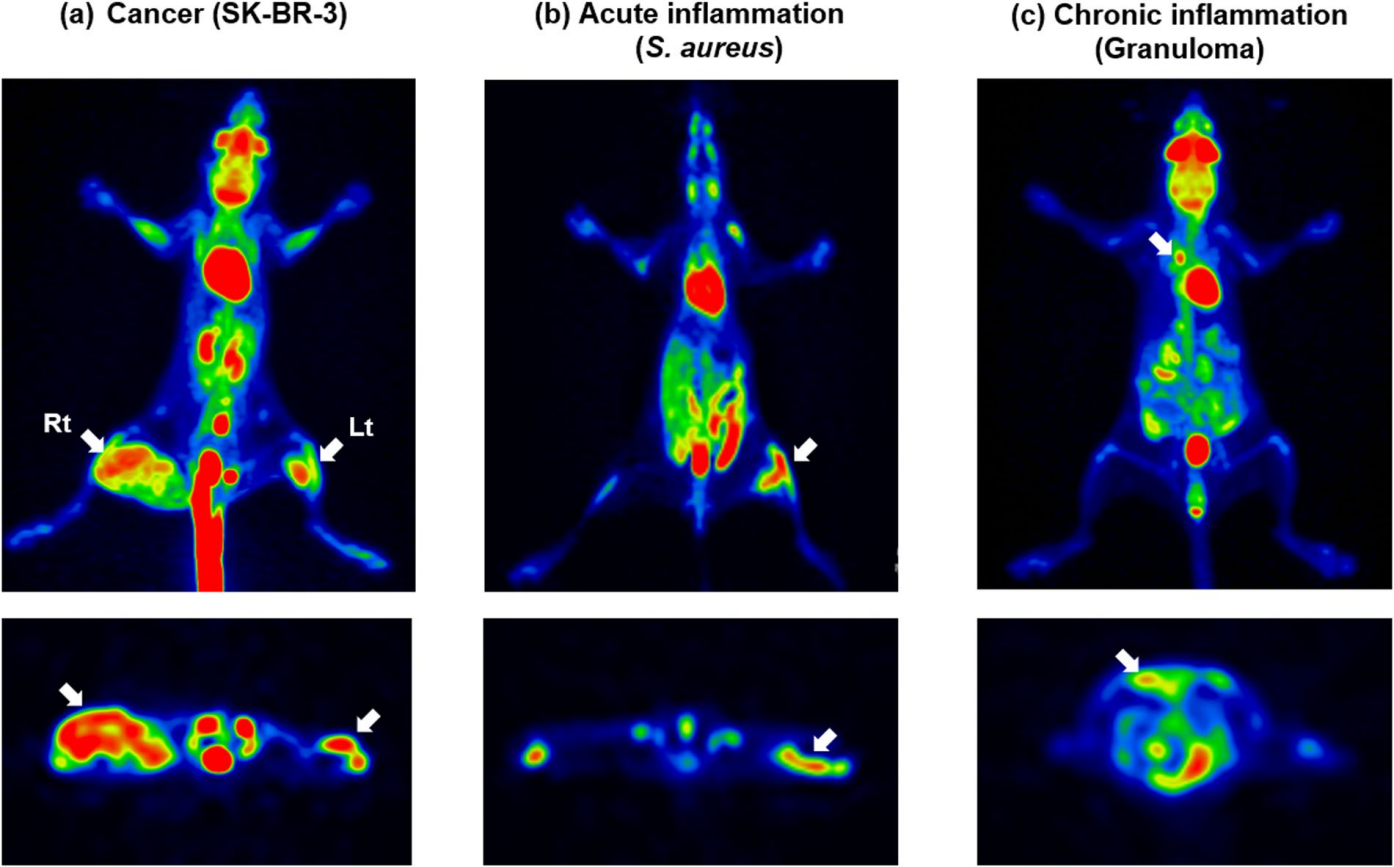
^18^F-FDG-PET images obtained from (**a**) the SK-BR-3 tumor-bearing mouse, which was inoculated with 5×10^5^ and 1×10^7^ cells in the left (Lt) and right (Rt) flank regions, respectively, and from (**b**) the acute and (**c**) chronic inflamed regions in a mouse infected with *S. aureus* and a mouse with the induced granuloma, respectively.

Next, we investigated whether our capacitance imaging technique could be used to discriminate cancer from inflammation. For this purpose, tumor-bearing nude mice were prepared by injecting 5 × 10^5^ and 1 × 10^7^ SK-BR-3 cells subcutaneously into the right and left flank regions, respectively. On the other hand, inflamed mice were prepared by injecting *Staphylococcus aureus* (*S. aureus*) into the buttocks using 4 × 10^7^ colony forming units for acute inflammation or by injecting Freund’s complete adjuvant into the air pouch for chronic inflammatory granuloma using C57BL/6 J mice because nude mice cannot tolerate infections. Figure 4 shows ^18^F-FDG-PET images obtained from these mice. A high uptake was found in both cancer and inflammation regions, indicating that there is the possibility of obtaining false positive results in inflammation regions^15–17^.

**Figure 5:**
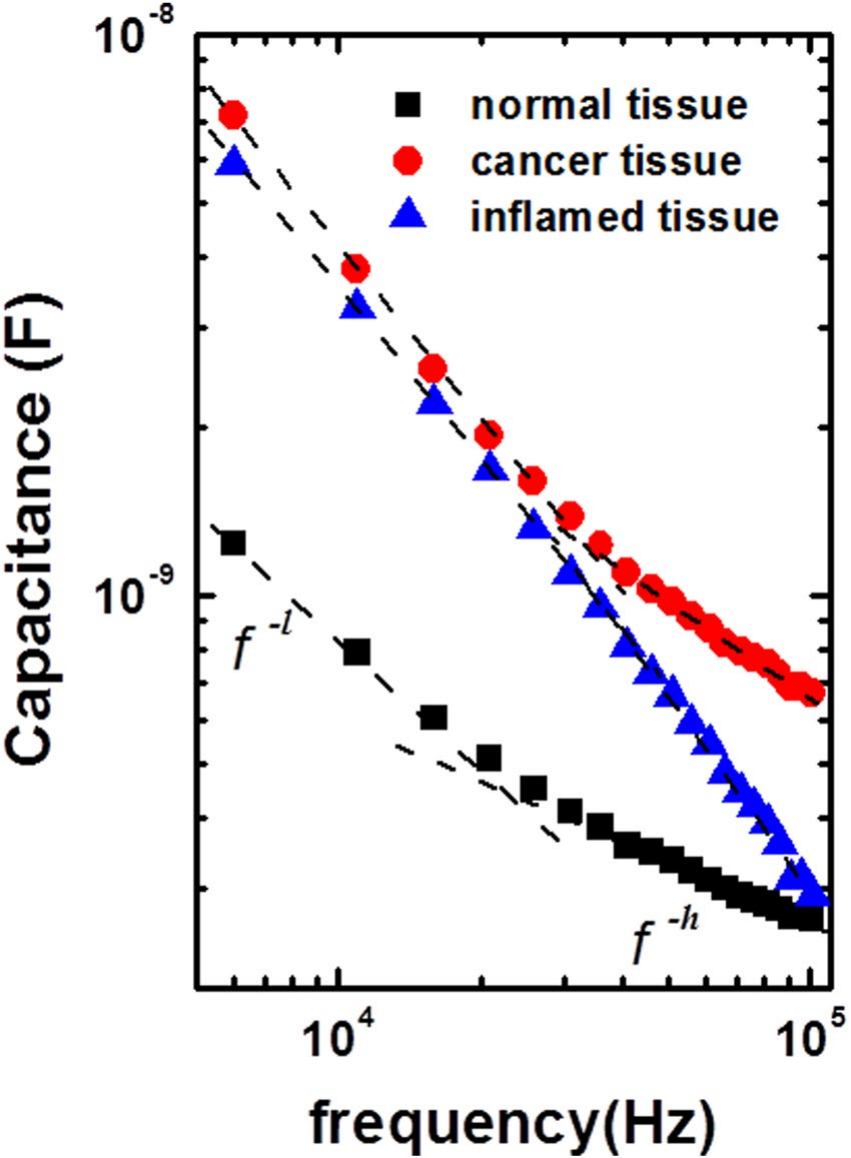
Frequency dependence of the capacitance measured for the normal and cancer tissues of the SK-BR-3 tumor-bearing mouse, and the inflamed tissue of the *S. aureus*-inoculated mouse. The data are fitted to the relationship *C* ∝ *f* ^−α^ with two different exponents at low frequencies (*f* ^−*l*^) and high frequencies (*f* ^−*h*^). For the normal and cancer tissues, *l* is larger than *h*, whereas for the inflamed tissue, *h* is larger than *l*.

Figure 5 shows the frequency dependence of the capacitance measured using probe I for the normal or cancer region of an SK-BR-3 tumor-bearing mouse and for the inflamed region of a mouse inoculated with *S. aureus*. A relationship of *C* ∝ *f* ^*−α*^ was observed for all tissues with two different exponents at low frequencies (*α = l*) and high frequencies (*α* = *h*) (*C*: capacitance and *f*: frequency). However, we noted that the value of *l*/*h* was different for inflamed tissue compared with normal or cancer tissue. For the normal or cancer tissue, *l*/*h* was > 1, whereas for the inflamed tissue, *l*/*h* was < 1. Furthermore, the capacitance of the inflamed tissue was higher than that of the normal tissue and lower than that of the cancer tissue over the entire frequency range. Accordingly, the low-frequency capacitance of the inflamed tissue was close to the capacitance of the cancer tissue, but the high-frequency capacitance of the inflamed tissue approached that of the normal tissue. In addition, we measured the frequency dependence of the capacitance at different body temperatures to investigate whether the values of *α* are dependent on temperature (Fig. S3). The body temperatures of anesthetized mice were maintained at about 37 °C when they were placed on an animal plate, while their body temperatures decreased to about 34 °C in 20 min when they were dismounted from the animal plate. For all normal, cancer, and inflamed tissues, the higher capacitance values were found at 34 °C as opposed to 37 °C because the dielectric constant of a solution usually increases with decreasing temperature^18^. However, the values of *α* were nearly unchanged, implying that the capacitance images may not be affected by the body temperature as long as the body temperature is maintained at the constant temperature.

**Figure 6:**
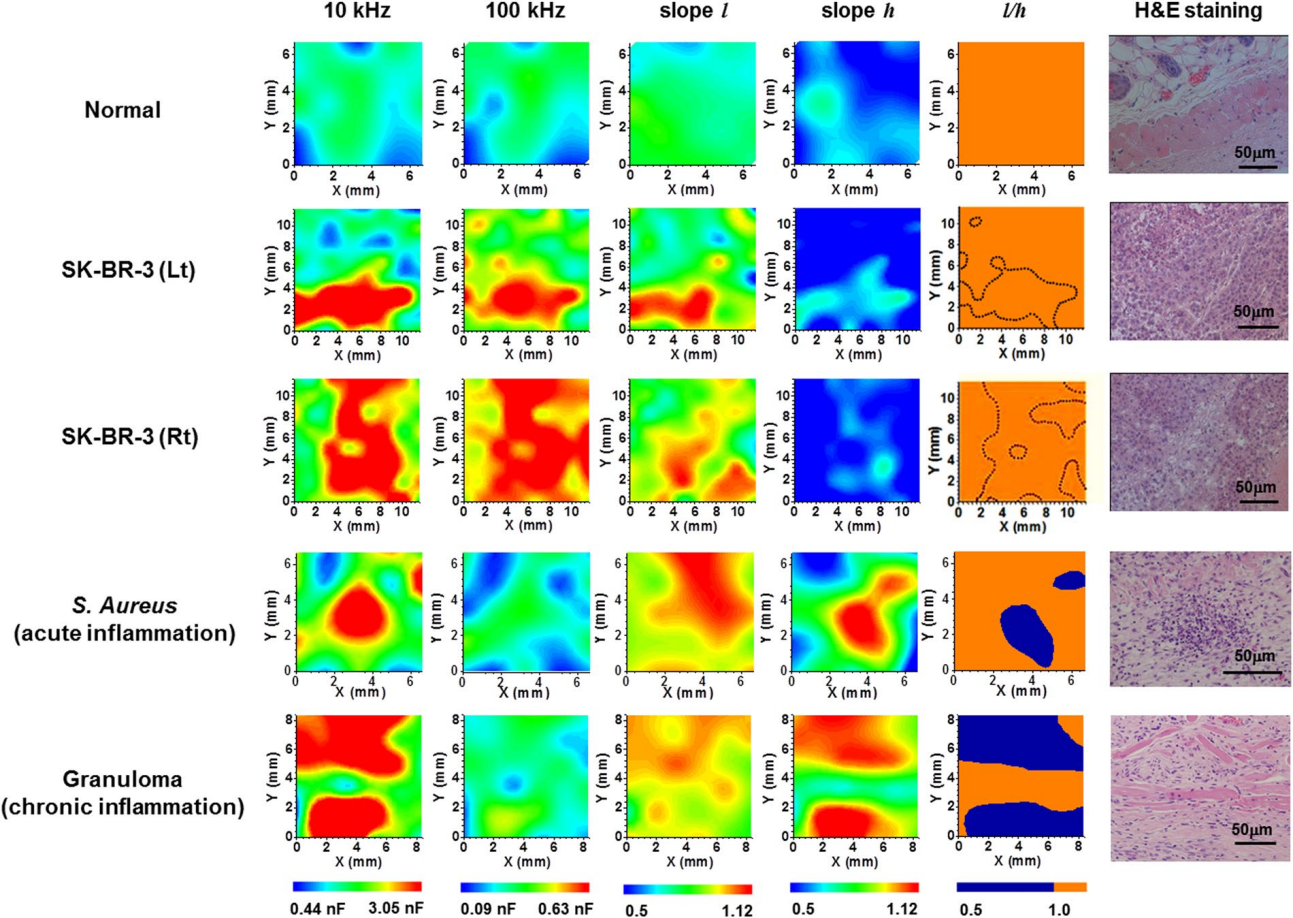
The capacitance images measured at 10 and 100 kHz, the maps of *l*, *h*, and *l*/*h*, and the H&E-stained histological specimens for a normal region, the left (Lt) and right (Rt) cancer regions of the SK-BR-3 tumor-bearing mouse, and the acute and chronic inflamed regions in a mouse infected with *S. aureus* and a mouse with the induced granuloma. The color range was set between the minimum capacitance of the control mouse (*C*_*normal*_) and 7 × *C*_*normal*_ at each frequency. The capacitance images were obtained from the same mice used to acquire the ^18^F-FDG-PET images (Fig. 4). The values of *l* and *h* were estimated from the plots of log(*C*) versus log (*f*) at low and high frequencies, respectively. In the map of *l*/*h*, *l*/*h < *1 is denoted by blue color and *l*/*h* > 1 by orange color, and the dotted region represents the red color region in the capacitance image at 100 kHz, which corresponds to the cancer region.

These observations suggested that cancer and inflammation may be distinguished by measuring the capacitance images at different frequencies. Hence, the capacitance images were acquired at 10 and 100 kHz for the normal and cancerous regions of the SK-BR-3 tumor-bearing mouse, as well as the acute and chronic inflamed (granuloma) regions of mice that were injected with *S. aureus* and croton oil in Freund’s complete adjuvant (CO/FCA), respectively (Fig. 6). The tumor-bearing mice exhibited similar capacitance images at 10 and 100 kHz. However, for the *S. aureus* or CO/FCA inoculated mice, different images were noted at 10 and 100 kHz; the red region seen at 10 kHz disappeared at 100 kHz owing to the rapid decrease in capacitance at high frequencies. The color maps of *l*, *h*, and *l*/*h*, which were estimated from the measured frequency dependence of the capacitance, are also included in Fig. 6. As expected, *l*/*h* > 1 (orange color) was observed for the normal and tumor tissues, while *l*/*h < *1 (blue color) was observed for the acute and chronic inflamed tissues. The blue regions in the map of *l*/*h* nearly overlapped with the red regions in the capacitance image at 10 kHz, suggesting that the blue regions in the *l*/*h* map may correspond to the inflamed regions. Additional experiments performed with a DLD-1 (a human colon cancer cell line) tumor-bearing mouse, another granuloma inoculated mouse, and a control mouse showed similar results (Fig. S4), confirming that cancer and inflammation may be differentiated using the capacitance images at different frequencies. The number of mice used for tumor and inflammation animal models is given in Table S1.

### Real-time monitoring of chemotherapeutic effects

**Figure 7:**
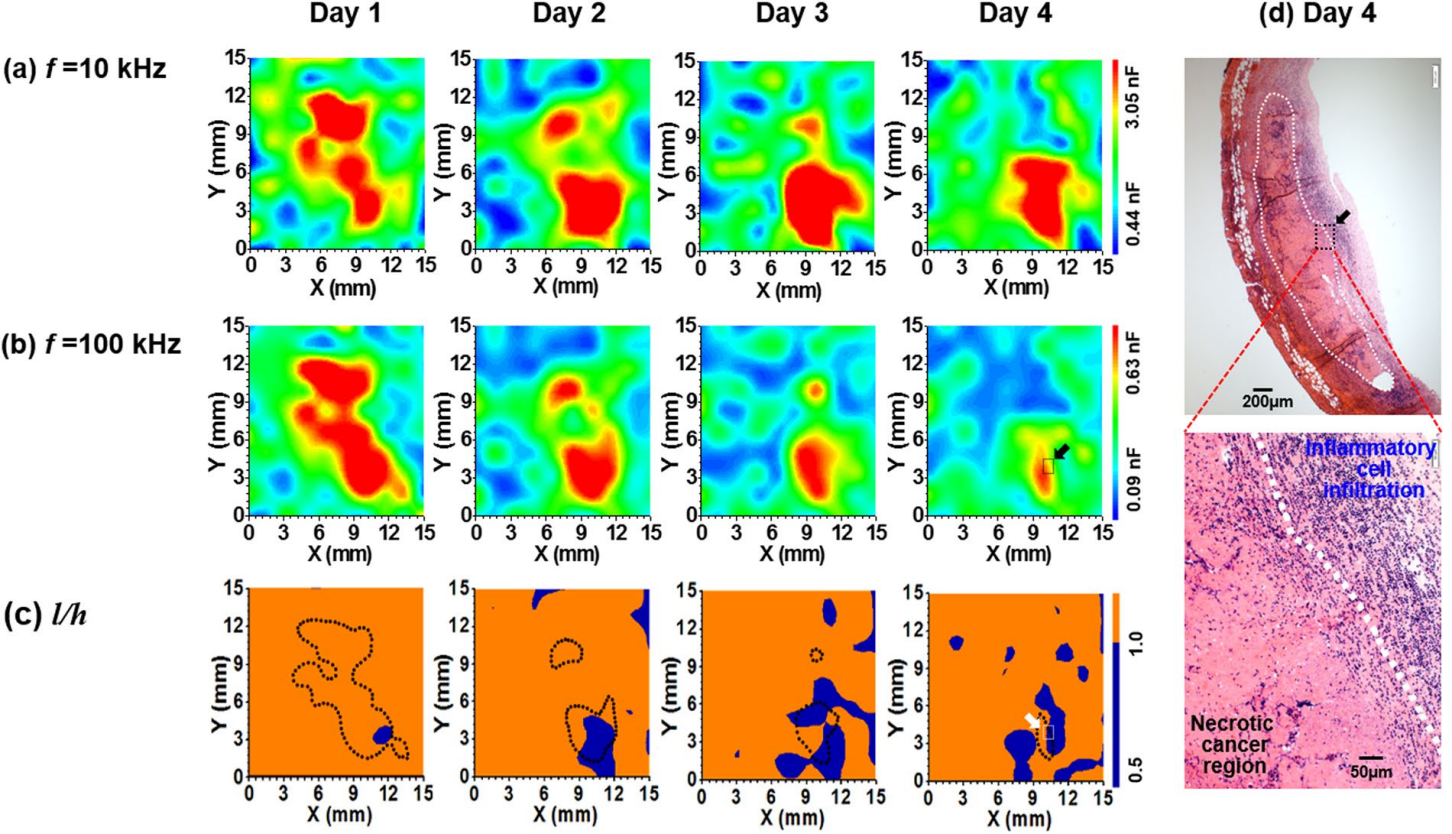
Time-lapse capacitance images measured at (**a**) 10 and (**b**) 100 kHz, and (**c**) the time-lapse maps of *l*/*h* for the cancer region of the SK-BR-3 tumor-bearing mouse treated with 100 μg/ml of DOX. The values of *l* and *h* were estimated from the measured frequency dependence of the capacitance. In the map of *l*/*h*, *l*/*h < *1 is denoted by blue color and *l*/*h* > 1 by orange color, and the dotted region represents the red color region in the capacitance image at 100 kHz, which corresponds to the cancer region. (**d**) H&E-stained specimen of cancer tissue extracted from the DOX treated SK-BR-3 tumor-bearing mouse on day 4. The white dotted area corresponds to the cancer region and the inset shows the enlarged histopathological image of the area denoted by an arrow. The black and white arrows in (**b**) and (**c**) are the same cancer region for histopathologic staining in (**d**).

To investigate whether the chemotherapeutic effects of an anti-cancer drug can be monitored in real-time, we measured the time-lapse capacitance images at 10 and 100 kHz for the cancer region of an SK-BR-3 tumor-bearing mouse treated with doxorubicin (DOX; 10 mg/kg, intratumoral injection) (Fig. 7). The tumor region was marked using micropore surgical tape (3 M, NDC 8333-1533-01) with printed dots at intervals of 0.5 mm (Fig. S1a), so the same region could be imaged when the measurements are repeated. On day 1, the sizes of the red regions were similar at 10 and 100 kHz, which is consistent with the red region representing the cancer tissue. However, as the size of the cancer region was reduced over time because of DOX treatment, the size of the red region decreased more rapidly at 100 kHz than at 10 kHz (Fig. 7a,b), and the size of the blue region increased in the color maps of *l/h* (Fig. 7c). These findings implied that some of the treated cancer tissues decreased in volume and became necrotic with infiltration of inflammatory cells of neutrophils, which was consistent with the histological findings (Fig. 7d). To confirm these findings, similar experiments were carried out in SK-BR-3 tumor-bearing mice, one untreated and another treated with DOX. In the DOX-untreated mouse, the red region at 100 kHz showed the tumor tissue, and the blue region in the color map of *l*/*h* was not observed at all for 4 days (Fig. S5a). In contrast, the red regions at 100 kHz were decreased when DOX was treated and the blue region in the color map of *l*/*h* increased over time in the DOX-treated mouse (Fig. S5b), which is consistent with the results shown in Fig. 7. These results verify that the chemotherapeutic effects can be monitored in real-time and without labels. In addition, the cancer region could be discriminated from the inflammatory region non-invasively using this capacitance imaging technique.

## Discussion

According to the dielectric model^19,20^, the dielectric constant in the linear regime of small electric fields is approximated as follows:

1 $${\rm{\varepsilon }}\approx \frac{9V{\varepsilon }_{e}^{2}}{16}{\chi }^{2}{a}^{2}\frac{{({R}^{+}-{R}^{-})}^{2}{S}^{2}}{{(R+2)}^{2}}\,$$

where *V* is the volume fraction occupied by the cells, *a* is the radius of the cells, *ε*_e_ is the permittivity of the electrolyte solution, and the other parameters, i.e., *χ*, *R*, *R* ^+^, *R* ^−^, and *S*, are the functions of zeta potential (*ξ*), as described in the Supplementary Note. At low frequencies, the electric current mostly passes through the extracellular space^21^, so the cell membranes act as insulators between extracellular and intracellular fluids, thus forming capacitors. Histopathologically, compared to normal tissues, the cell density is higher in cancer tissues because of cell proliferation or in inflamed tissues because of bacterial growth or immune cell infiltration. Therefore, *V* is expected to be larger for cancer or inflamed tissues than for normal tissues, resulting in a higher capacitance. The lower capacitance observed in the cancer region with more necrotic cells (Fig. 3b) is also explained by Eq. (1) as the cell membranes are ruptured by necrosis, leading to a decrease in *V*.

However, at high frequencies, the cell membranes have negligible impedance, so the current penetrates the cell membrane and flows within the intracellular space^22,23^. Thus, the capacitance at high frequencies may be governed by *ε*_e_ rather than *V*. Compared to normal cells, cancer cells have higher water content and sodium concentration^24^, and utilize anaerobic glycolysis to compensate for their faster metabolism (Warburg effect^3,25^). As a result, the *ε*_e_ of cancer tissue may be higher than *ε*_e_ of normal tissue, which explains the higher capacitance values in cancer tissues. On the other hand, inflamed tissue is composed of normal leukocytes and exudate, unlike the cancer tissue; thus, the high-frequency capacitance of the inflamed tissue approaches that of normal tissue.

^18^F-FDG PET is widely used in cancer diagnosis and therapeutic monitoring. However, ^18^F-FDG is not a tumor-specific substance, and its accumulation may be observed in acute and chronic inflammatory lesions, resulting in false positive findings;^16,17^ therefore, diagnostic MRI, CT, or US images are sometimes combined. In our study,^18^F-FDG positive cancer and inflamed tissues may be diagnosed and differentiated by dielectric imaging at different frequencies. The dielectric imaging system can be easily applied to the skin or mucosal surface area where the probe is easily accessible, and it is advantageous to monitor the growth of cancer in real-time without the need for radioisotope tracers, although it has limitations with respect to detecting the mass in deep tissues or in the brain region. Further development of dielectric imaging systems combined with fibroscopic instruments is necessary to diagnose a mass inside the internal body, such as the gastrointestinal tract, intraperitoneal, or nasopharyngeal regions.

In summary, we have developed an *in vivo* dielectric imaging technique using pin-type electrodes on a flexible substrate. Compared with normal tissues, cancer tissues exhibited higher capacitance values, so cancer may be imaged by measuring the capacitance values in the cancer region, which was confirmed by making comparisons with histopathological results. To investigate whether inflammation can be discriminated from cancer with our imaging technique, the capacitance images were acquired at different frequencies for cancer and inflamed regions. Cancer and inflammation showed different images at different frequencies, indicating that cancer and inflammation may be non-invasively distinguished with this imaging technique, although further studies are necessary on various conditions of cancer, for example, different metabolic activities or proliferation rates, and various bacterial infections. In addition, we have demonstrated that the *in vivo* dielectric imaging technique may be applied to monitor chemotherapeutic effects in real-time and without labels.

## Methods

### Construction of probes

We constructed two types of probes for the *in vivo* capacitance measurements. The first one was a pin-type probe (probe I, Fig. 1a) that was made from stainless steel wires 200 μm in diameter. The tips of the wires were sharpened to a needle shape; thus, they easily penetrated into the mouse skin with minimal bleeding or damage. Two pin electrodes, which were 1.5 mm in length and spaced 0.5 mm apart, were fixed to a trapezium-shaped plastic structure to maintain the spacing between the two electrodes, and they were electrically connected to printed circuit boards (PCBs) using conductive silver epoxy. The other probe was a 10 × 10 pin-type (probe II, Fig. 1b) with pin electrodes spaced 0.5 mm apart. The 10 × 10 pin electrodes were aligned in a custom-made flexible plastic structure that had springs on each side. Most of the pin length (1.5 mm) was exposed outside the channel structure, while 0.5 mm was hidden inside the channel structure to move back and forward on the mouse skin. The pin array was electrically connected to a PCB using conductive silver epoxy, and then the PCB was connected to a high-definition multimedia interface plug terminal.

### Ethical approval

Animal studies were performed according to the Guide for the Care and Use of Laboratory Animals (National Research Council, USA), and experimental procedures in this study were approved by the Institutional Animal Care and Use Committee of Yonsei University Health System (YUHS, Approval No. IACUC2014-0008-4). All mice were maintained in a specific pathogen-free facility at the YUHS. The animals had access to food and water *ad libitum*.

### Preparation and handling of animal models

#### Tumor formation

Female BALB/c nude mice (8 weeks old, ~20 g) were supplied by Orient Bio Inc. (Seongnam-si, Korea). For tumor induction, SK-BR-3, MCF-7, A431, or DLD-1 cells were harvested, and 5×10^5^ or 1 × 10^7^ viable cells were injected subcutaneously into the flank region of the mice. Tumors were grown for 2–4 weeks before *in vivo* capacitance measurements. The size of the tumor was measured using a caliper and the tumor volume was estimated by the length × width × height × π/6^26^.

#### *S. aureus* infection

For the acute inflammation model, C57BL/6 J mice (8 weeks old) were obtained and Gram-positive *S. aureus* (4 × 10^7^ colony forming units in 20 μl) was injected at a depth of 2–4 mm into the buttock with a 29 1/2-G hypodermic needle^27^. For the control, 20 μl of phosphate-buffered saline (PBS) was injected into the other side. Capacitance measurements were conducted after 24 h.

#### Granuloma

For the chronic inflammation model, granuloma was generated in C57BL/6 J mice (8 weeks old) using a chronic air pouch^28^. The air pouch was raised on the dorsum of the mouse by subcutaneous injection of 3 ml of sterile air, and 0.1 ml of 0.5% croton oil in Freund’s complete adjuvant (CO/FCA) was injected into the air pouch. Capacitance measurements were conducted 6-10 days later.

### Capacitance measurements

For *in vivo* capacitance measurements, probes I and II were sterilized using 70% ethanol. Then, the capacitance was measured with an LCR meter (Agilent 4284 A) with a 10-mV peak-to-peak AC voltage over a frequency range of 1 ~ 100 kHz. The capacitance measurements with probe II were carried out using a data acquisition/switching unit (Agilent 34970 A) connected to the LCR meter. Data were measured at each point three times and then averaged. While the capacitance was measured, the mouse was anesthetized with Zoletil50/Rompun and positioned at an animal plate controlled at 37 °C. To monitor the therapeutic effect of an anti-cancer drug, doxorubicin (10 mg/kg) was injected intratumorally. Then, the capacitance images were obtained for 5 days. For real-time monitoring of chemotherapeutic effects, the micropore surgical tape (3 M, NDC 8333-1533-01) printed the 10×10 capacitance array was attached onto the tumor site to keep the array position (Fig. S1a). The 9 × 9 capacitance values were plotted on a 50-color scale using the software OriginPro® version 9 (Northampton, MA. USA).

### Histopathology

The mice were anesthetized and sacrificed after the *in vivo* capacitance measurements. Portions of tumor or inflamed tissues were removed for histopathological examination, and fixed in 10% buffered formalin-saline at 4 °C overnight. Then, tissues were embedded in paraffin blocks and 4-μm-thick sections were mounted on a glass slide for hematoxylin and eosin (H&E) staining. After washing in PBS, the secondary antibody (DAKO EnVision Rabbit/Mouse Kit, DAKO) was applied for 30 min at 25 °C, and then the section was developed with diaminobenzidine, and counterstained with hematoxylin.

### Micro-PET

PET scanning was performed using the Siemens Inveon small animal PET scanner (Siemens Medical Solutions). Anesthesia was administered with 2.5% isoflurane and maintained for 50 min of the PET experiment with 1.5% isoflurane. After cannulation in a tail vein, mice were positioned in the center of the gantry. Tracer accumulations in the SK-BR-3 tumor-bearing and *S. aureus*-bearing mice were investigated by performing dynamic PET scans over 180 min after the injection of ^18^F-FDG.

## Electronic supplementary material

Supplementary information
