# Supplementary material for: Dielectric imaging for differentiation between cancer and inflammation in vivo
Source: Sci Rep. 2017 Oct 13;7:13137. doi: 10.1038/s41598-017-13545-3 (PMC5640678; doi:10.1038/s41598-017-13545-3)
Supplement: Supplementary file 1 — Supplementary information [file 41598_2017_13545_MOESM1_ESM.pdf]

Supplementary information

Dielectric imaging for differentiation between cancer and inflammation *in vivo*

Rimi Lee[1+], Sun-Mi Lee[1+], Hyung Joon Kim[1], Sook Young Kim[2,3], Mina Son[1], Jun Ho Song[4], Khulan Lkhamsuren[2], In Ho Park[2,5], Inhong Choi[2], Young Nyun Park[3], Jeon-Soo Shin[1,2,5*], Kyung-Hwa Yoo[1,4*]

[1]Graduate Program for Nanomedical Science and Technology, Yonsei University, Seoul, 03722, Republic of Korea.

[2]Department of Microbiology, Yonsei University College of Medicine, Seoul, 03722, Republic of Korea.

[3]Department of Pathology, Yonsei University College of Medicine, Seoul 03722, Korea.

[4]Department of Physics, Yonsei University, Seoul, 03722, Republic of Korea.

[5]Severance Biomedical Science Institute and Institute for Immunology and Immunological Diseases, Yonsei University College of Medicine, Seoul 03722, Republic of Korea.

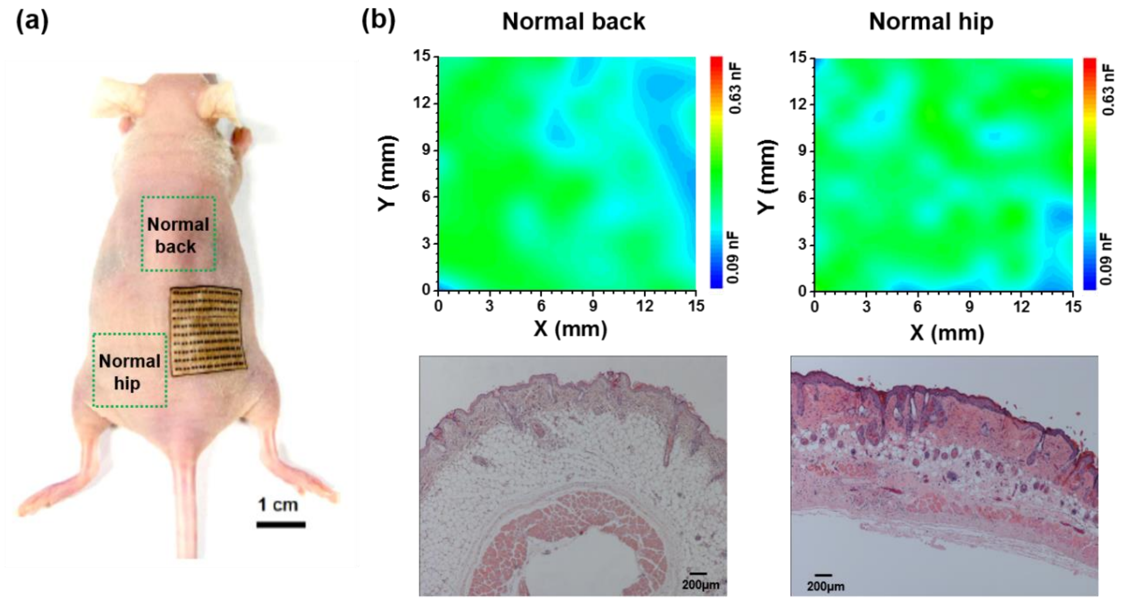

Figure S1. (a) Mouse regions for capacitance images. (b) (Top) Capacitance images of normal back and hip regions of the mouse on a 50-color scale with the red color denoting the highest capacitance. A 9 × 9 capacitance array was measured using probe II with an AC voltage of 10 mV at 100 kHz. (Bottom) H&E-stained histological specimens of normal tissues extracted from the back and hip regions after the capacitance measurements. No apparent damage was detected after the capacitance measurements.

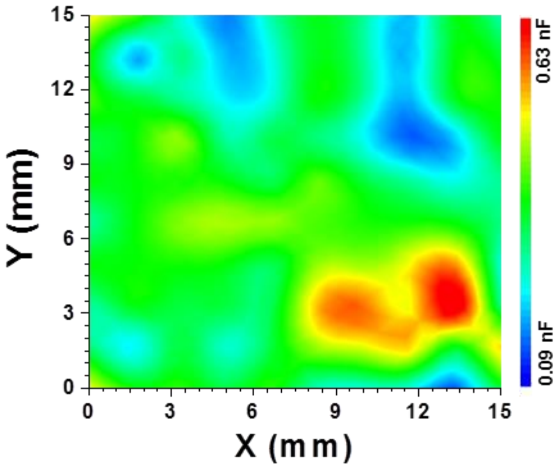

Figure S2. Capacitance image of the cancer region obtained from the SK-BR-3 tumor-bearing mouse using 9 × 9 capacitance array.

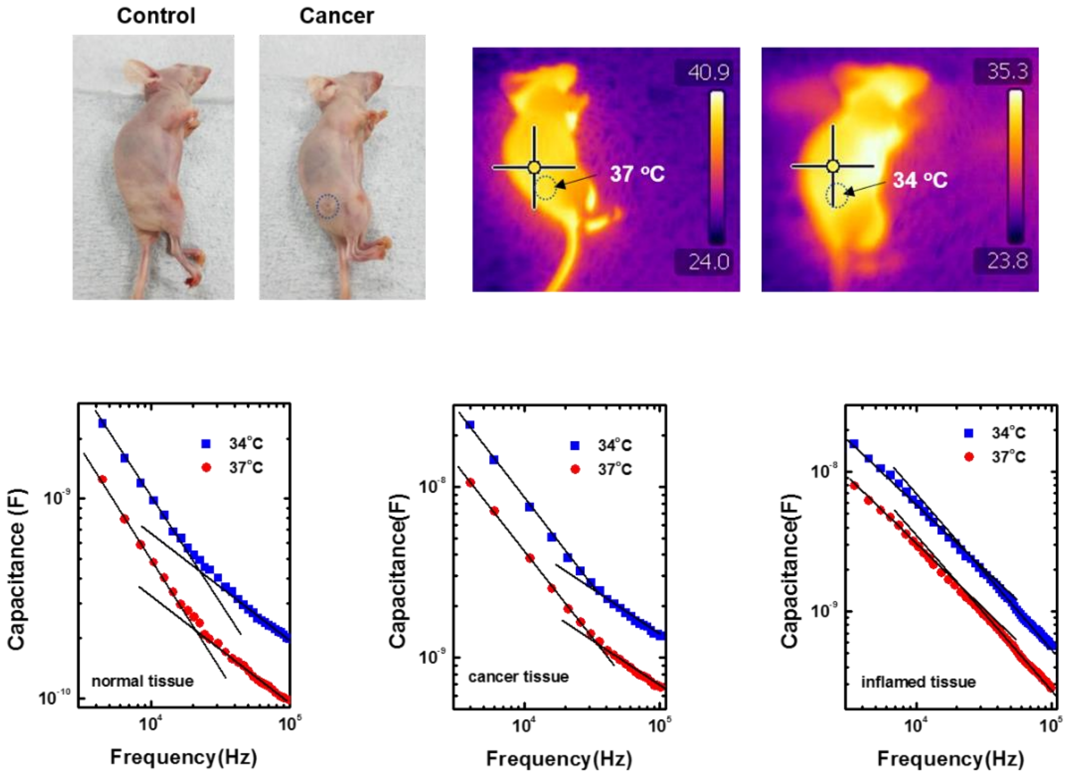

Figure S3. Frequency dependence of the capacitance measured for the normal and cancer tissues of the SK-BR-3 tumor-bearing mouse, and the inflamed tissue of the *S. aureus*-inoculated mouse with two different temperature at 34 and 37°C. The body temperature of mice was identified by the thermal imaging camera (FLIR-T250, FLIR, Sweden).

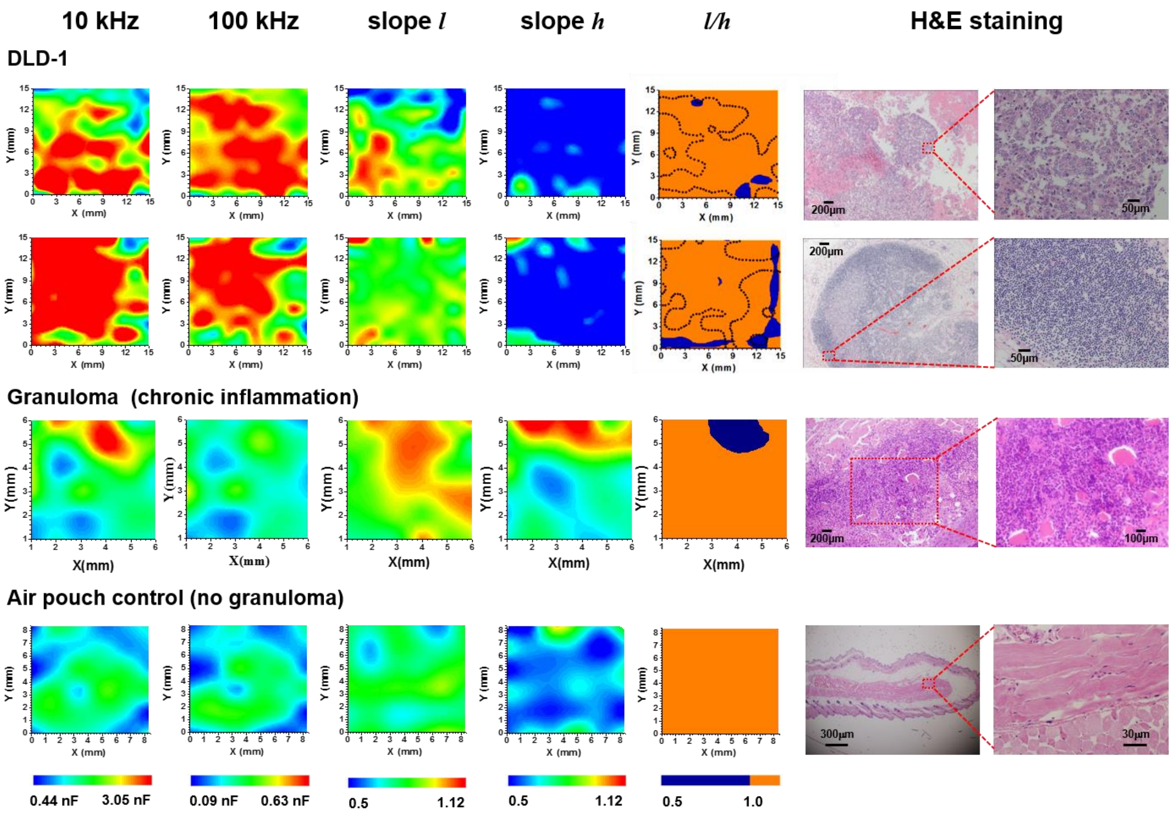

Figure S4. The capacitance images measured at 10 and 100 kHz; the maps of *l*, *h*, and *l*/*h*; and H&E-stained specimens for the cancer region of a DLD-1 tumor-bearing mouse, the inflamed region of a mouse infected with the induced granuloma, and the air pouch control mouse. The color range was set between the minimum capacitance of the control mouse (C_{normal}) and 7 × C_{normal} at each frequency. The values of *l* and *h* were estimated from the plots of log(*C*) versus log(*f*) at low and high frequencies, respectively. In the map of *l*/*h*, $l/h < 1$ is denoted by blue color and $l/h > 1$ by orange color, and the dotted region represents the red color region in the capacitance image at 100 kHz, which corresponds to the cancer region.

(a) DOX untreated SK-BR-3 tumor-bearing mouse

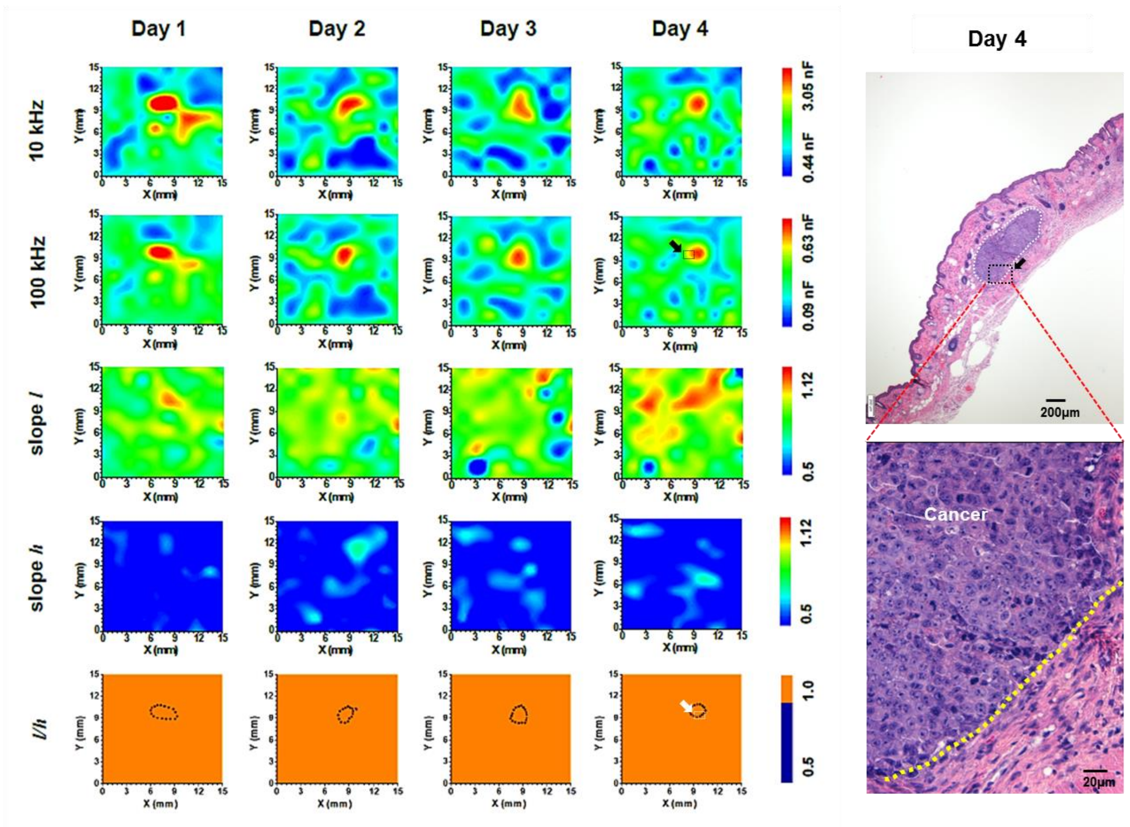

(b) DOX treated SK-BR-3 tumor-bearing mouse

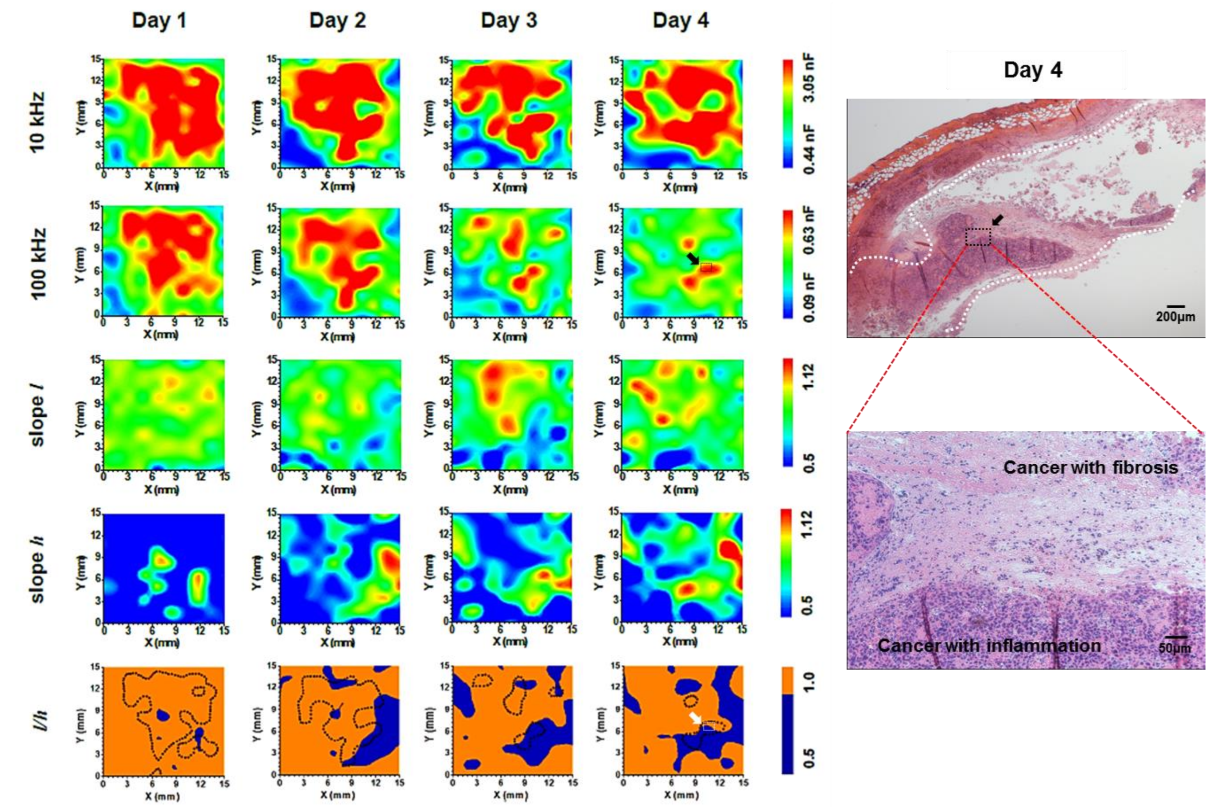

Figure S5. Time-lapse capacitance images measured at 10 and 100 kHz, and the time-lapse maps of *l*, *h*, and *l*/*h* for the cancer regions of an untreated (a) or DOX treated (b) SK-BR-3 tumor-bearing mouse. The values of *l* and *h* were estimated from the measured frequency dependence of the capacitance. In the map of *l*/*h*, the dotted region represents the red color region in the capacitance image at 100 kHz, which corresponds to the cancer region. The right panel shows the H&E-stained specimens of cancer tissues extracted from the untreated and the DOX treated SK-BR-3 tumor-bearing mouse at day 4. The white dotted area is the tumor region and the insets show the enlarged histopathological images of the area denoted by an arrow.

Supplementary Note

According to the dielectric model, the dielectric constant at low frequencies in the linear regime of small electric fields is approximated as:

$$\varepsilon \approx \frac{9V\varepsilon_e}{16}\chi^2 a^2 \frac{(\mathrm{R}^+ - \mathrm{R}^-)^2 \mathrm{S}^2}{(\mathrm{R}+2)^2}$$

where

$$\chi = \sqrt{\frac{2\sigma_e}{(D^+ + D^-)\varepsilon_e}}$$

$$R^\pm = \frac{4}{\chi a}\left(exp\left(\mp\frac{e\xi}{2kT}\right) - 1\right)(1+3m^\pm) \pm \frac{6m^\pm e\xi}{\chi akT}$$

$$m^\pm = \frac{2\varepsilon_e}{3\eta D^\pm}\left(\frac{kT}{e}\right)^2$$

$$R = \frac{D^+R^+}{D^+ + D^-} + \frac{D^-R^-}{D^+ + D^-}$$

$$S = \frac{\dfrac{2(R+2)}{(R^+ + 2)(R^- + 2)}}{1 - P\dfrac{R+2}{(R^+ + 2)(R^- + 2)}}$$

$$P = \frac{D^+ + D^-}{2D^+D^-}\frac{48D^\pm m^\pm}{\chi a}\ln\left[\cosh\left(\frac{e\xi}{4kT}\right)\right]$$

Here, V is the volume fraction occupied by the cells; a is their radius; ε_e is the permittivity of electrolyte solution, $\mathrm{D}^\pm$ are the diffusion coefficients of counterions and co-ions, ξ is the Zeta potential, and η is the viscosity. The electrolyte solution conductivity, σ_e, and the permittivity of electrolyte solution, ε_e, are considered to be independent of the frequency at low

frequencies. Other constants include e = 1.602 × 10^{-19} C, k = 1.381 × 10^{-23} J/K, T = 310 K and ε_e= 78.5 × 8.85×10^{-12} C^2/Nm^2.

Table S1. The prepared tumor and inflammation animal models in this study.

	Cancer cell/Infection	Treatment	No.
Tumor	SK-BR-3	-	13
		Doxorubicin (10 mg/kg)	10
	MCF-7	-	5
	A431	-	5
	DLD-1	-	5
Acute inflammation	*S. aureus*	-	5
Chronic inflammation	Granuloma (CO/FCA)	-	3

SK-BR-3 and MCF-7: human breast cancer cell lines, A431: human epidermoid carcinoma cell line, DLD-1: human colon cancer cell line, *S. aureus*: *Staphylococcus aureus*, CO/FCA: croton oil/Freund's complete adjuvant.
